# Genetic associations between ULK3 and epilepsy: a two-sample Mendelian randomization study

**DOI:** 10.3389/fneur.2024.1376314

**Published:** 2024-08-12

**Authors:** Baolai Liu, Keyi Fan, Xinyi Zheng, Yaochen Zhang, Shangkai Bai, Zhentong Liu, Shuhan Xu, Zhihao Su, Huiting Cao, Heyi Zhang, Shengxiao Zhang

**Affiliations:** ^1^Department of Neurosurgery, Shanxi Provincial People's Hospital, The Affiliated People’s Hospital of Shanxi Medical University, Taiyuan, China; ^2^Key Laboratory of Cellular Physiology at Shanxi Medical University, Ministry of Education, Taiyuan, Shanxi, China

**Keywords:** ULK3, Mendelian randomization, epilepsy, causality, genetics

## Abstract

**Background and objectives:**

Observational studies have suggested that a multitude of pathological processes and biomolecules are involved in the initiation and development of epilepsy, and ULK3 is linked to the nervous system. However, it remains uncertain whether this association between ULK3 and epilepsy is causal and the direction of any causal relationship. This study employs a two-sample Mendelian randomization (MR) method to investigate the relationship between ULK3 and the risk of epilepsy.

**Methods:**

We analyzed genome-wide association study (GWAS) summary statistics for ULK3 (sample size = 3,301), focal epilepsy (sample size = 39,348), and generalized epilepsy (sample size = 33,446). Bidirectional MR analyses were conducted to explore these relationships. We selected a set of single nucleotide polymorphisms (SNPs) with an association threshold of less than 1 × 10^−5^ as instrumental variables for further analysis. Various MR methods, including Inverse Variance Weighted, Weighted Median, MR-Egger Regression, Simple Model, Weighted Model, and Robust Adjustment Profile Score were used. Sensitivity analyses were performed to ensure the robustness of the results.

**Results:**

Our MR analyses revealed a causal relationship where an increased level of ULK3 was associated with a decreased risk of focal epilepsy (odds ratio = 0.92, 95% confidence interval: 0.86–1.00, *p* = 0.041). No significant heterogeneity (Q = 7.85, *p* = 0.165) or horizontal pleiotropy (Egger regression intercept = 0.0191, *p* = 0.415) was detected. However, in the reverse analysis, we found no significant causal effect of focal epilepsy on ULK3 (*p* > 0.05). Furthermore, no significant causation was identified between ULK3 and generalized epilepsy (*p* > 0.05).

**Conclusion:**

This study suggests a causal relationship between ULK3 and the risk of focal epilepsy from a genetic perspective. Nevertheless, further investigation is needed to understand the role of ULK3 in epilepsy fully.

## 1 Introduction

Epilepsy, one of the most prevalent and debilitating neurological disorders, is characterized by active, transient, reproducible, and often paroxysmal disruptions of the central nervous system (CNS) (1). It can be broadly classified into three groups based on genetic factors, underlying mechanisms, and clinical effects: focal epilepsy, generalized epilepsy, and unknown epilepsy, depending on the type of seizure. Focal epilepsy originates within networks limited to one hemisphere and in subcortical structures. It may be discretely localized or more widely distributed. In contrast, generalized epilepsy originates from some point within, involving bilateral cerebral cortex and subcortical structures, and rapidly spreads to bilaterally distributed networks (2). According to the Global Epilepsy Burden Report, approximately 130,000 individuals succumb to epilepsy-related complications annually, with half a million people worldwide diagnosed with epilepsy, resulting in 125,000 annual deaths (3). Understanding the causes and mechanisms underlying epilepsy is crucial to identifying novel opportunities for ameliorating the condition.

ULK3, a serine/threonine protein kinase located in the cytoplasm, plays a key role in regulating Sonic Hedgehog (SHH) signaling and autophagy (4). On the one hand, ULK3 can positively regulate the transmission of SHH signaling, as a regulator of the SHH signaling pathway that regulates various developmental processes, tissue homeostasis, and adult neurogenesis, in addition to processes such as protein autophosphorylation and regulation of the smoothened signaling pathway (5, 6). On the other hand, studies had demonstrated that ULK3 involved in autophagy as a positive regulator of GLI1 (GLI family zinc finger 1) (5). These findings highlight ULK3’s role in both autophagy and cell division processes. Autophagy dysfunction is associated with various human diseases, including cancer, heart disease, autoimmune disorders, and neurological conditions, such as epilepsy (7). In neurons, autophagy is dynamically regulated in different cellular compartments, including somatic cells, axons, and dendrites. Dysfunctional autophagy in epilepsy is primarily due to an imbalance between excitation and inhibition in the brain. Autophagy has been considered a potential target for neurological disease treatment (8). Consequently, we conducted this study to assess the causal relationship between ULK3 and epilepsy, with the aim of improving epilepsy treatment strategies.

Mendelian randomization (MR) is a method that utilizes genetic variants as instrumental variables (IVs) to determine whether a risk factor causally affects a health outcome (9). The Mendelian randomization is based on Mendel’s second law, the independent separation of genetic alleles when DNA is passed from parent to offspring during gametic formation (10). Mendelian randomization analysis can provide critical evidence for the potential causal effects of many modifiable exposures, including traditional epidemiological risk factors, lifestyle factors, and drug targets (11). It is designed to provide unbiased assessments of causality as much as possible (12). To address the gaps in our understanding of the relationship between ULK3 and epilepsy, we conducted a two-sample MR analysis to investigate their causal connection.

## 2 Methods

### 2.1 Data source

The genome-wide association study (GWAS) data for epilepsy, including generalized epilepsy (cases = 3,769) and focal epilepsy (cases = 9,671), was obtained from the 2018 International Alliance against Epilepsy (ILAE) Complex Epilepsy Alliance, consisting of 15,212 cases of epilepsy and 29,677 controls (13). The genetic variation data for ULK3 were sourced from the human plasma protein genome map,^1^ derived from a study of 3,301 healthy individuals, linking genetic variations to disease and drug databases (14).

### 2.2 Study design

**Figure 1:**
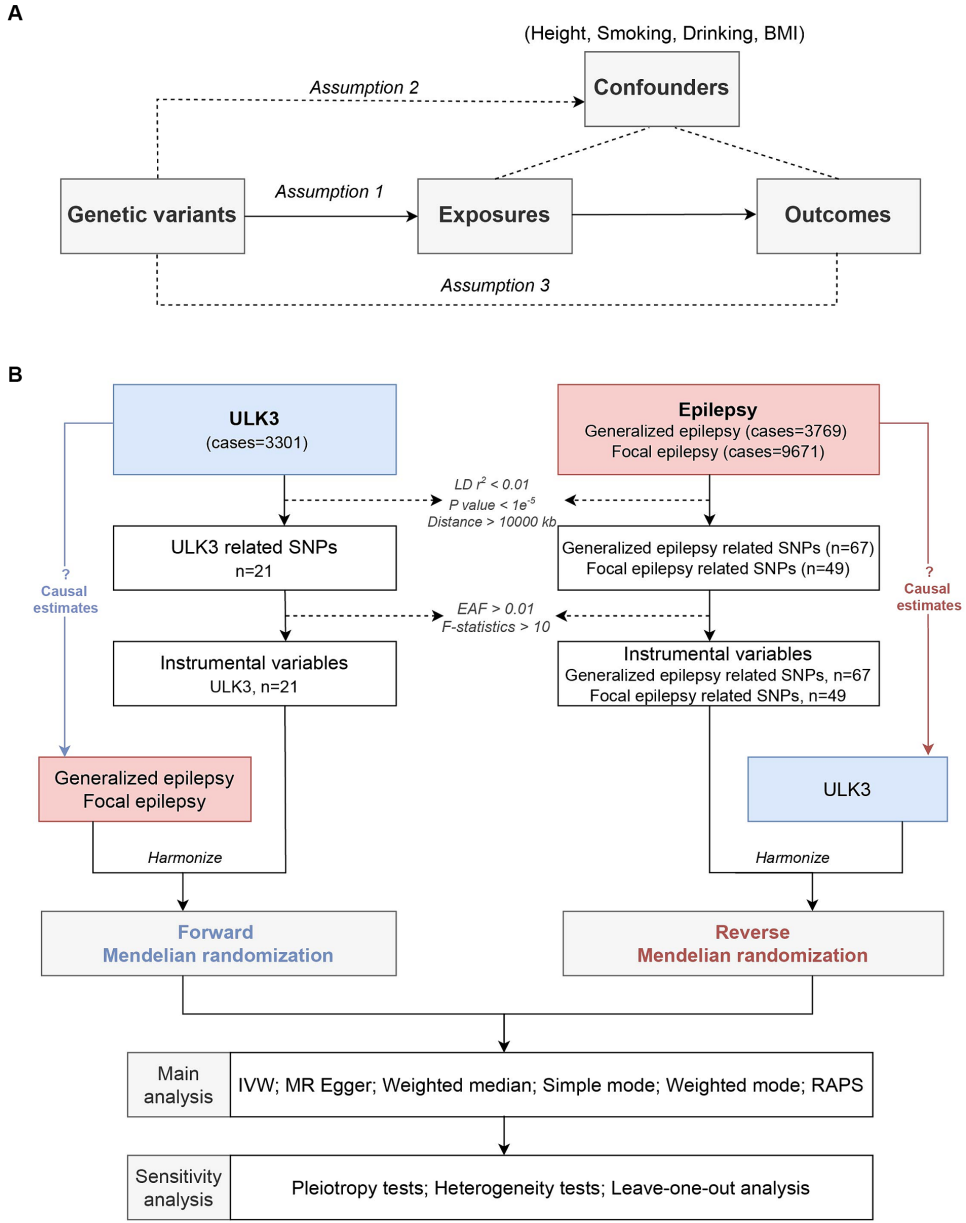
Study design. **(A)** Schematic diagram of the assumptions in Mendelian randomization (MR) model. **(B)** Flow diagram of this current MR framework.

We conducted a two-sample MR analysis using the GWAS data for epilepsy and the ULK3 data. To ensure the robustness of our results, we performed multiple sensitivity analyses. MR research necessitates meeting three core conditions: (1) IVs are closely related to ULK3; (2) IVs are independent of confounding factors; (3) IVs affect focal epilepsy and generalized epilepsy exclusively through ULK3 (Figure 1) (15).

### 2.3 Instrumental variable selection

We rigorously screened single nucleotide polymorphisms (SNPs), selecting those with a genome-wide significance threshold (*p* < 1e^−5^). To ensure the independence of IVs, we selected SNPs with an R^2^ of ≥0.01 within a 10,000 kb-sized window (16). We used PhenoScannerV2 to remove SNPs related to IVs (17), such as height (18), BMI (19), smoking (20), drinking (21), and other potential confounding factors. We calculated the F-statistic of IVs, selecting SNPs with *F* ≥ 10 and a minor allele frequency ≥ 0.01 to minimize the impact of weak IVs on bias (22). The remaining SNPs were used for MR analysis.

### 2.4 Statistical analysis

To assess the causal relationship between ULK3 and epilepsy, we performed MR analysis using the following methods: Inverse Variance Weighted (IVW): This method aggregates two or more random variables to minimize the sum variance and calculate the Wald ratio of the causal effect of each SNP (23). IVW results served as a primary basis, with other methods providing Supplementary Data Sheet 1 (24). MR-Egger Regression: This method fits regression models to test the existence of SNPs’ horizontal pleiotropy and quantifies SNPs with an intercept term. An intercept term of 0 indicates no horizontal pleiotropy (25). Weighted Median: This method provides a consistent and reliable causal estimate even when only half of the effective IVs are available (26). Simple Mode: Simple mode is not as powerful as IVW, but it provides robustness for pleiotropy. When the largest group of instruments with MR estimates is valid, Weighted Mode based causal estimation consistently estimates the causal effect mode (27). Robust Adjustment Profile Score (RAPS): This method is robust to system multiplicity and heterogeneity, reducing the influence of weak IVs and enhancing statistical effectiveness (28).

To ensure the robustness of our analysis, we conducted several sensitivity analyses: Cochran’s Q Statistics were used to evaluate heterogeneity among IVs (29). The intercept term of MR-Egger regression was calculated to assess horizontal pleiotropy (30). Leave-One-Out (LOO) Analysis was employed to eliminate SNPs one by one, evaluating whether the results of MR depended on a specific SNP (31).

The analysis was conducted using R (version 4.3.1) and the Two-sample MR analysis package (version 0.5.7). All datasets were analyzed in both forward and reverse directions, with a significance threshold of *p*-value < 0.05 applied.

## 3 Results

### 3.1 Selection of IVs

A total of 21 SNPs met the genome-wide significance threshold for IVs related to ULK3. After matching with the GWAS data for focal epilepsy and generalized epilepsy, six SNPs remained. None of these SNPs were associated with confounding factors in PhenoScanner, and all were employed in subsequent MR analysis. The F-statistic, with a value of 26.98, indicated the presence of strong instruments.

### 3.2 ULK3 and focal epilepsy

**Figure 2:**
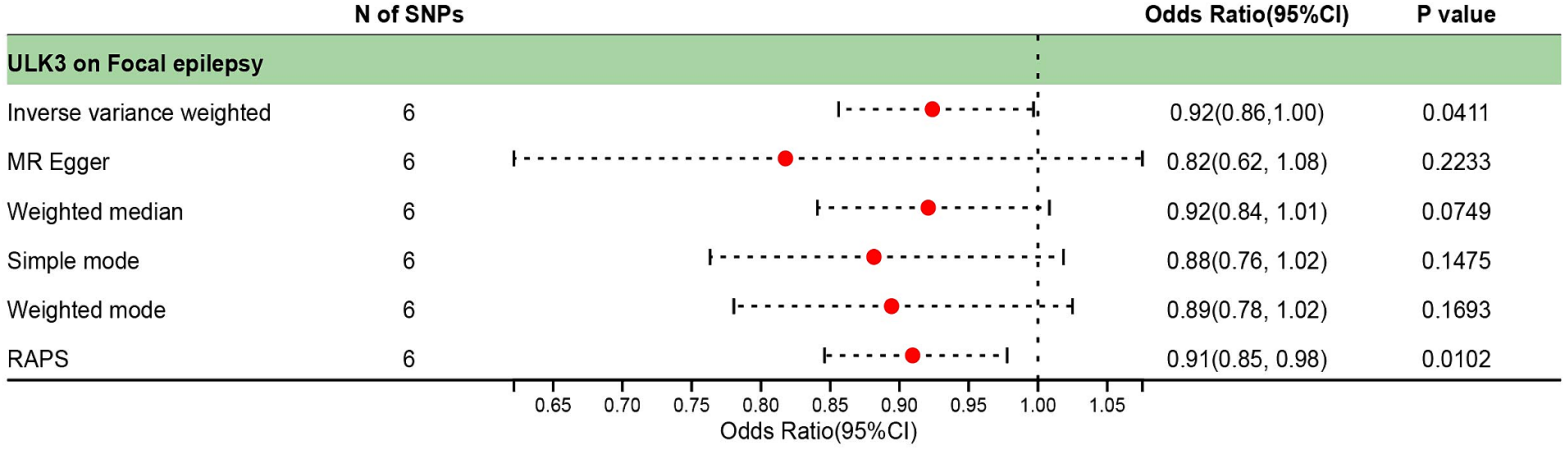
Forest plot of causal effect between ULK3 and focal epilepsy.

**Figure 3:**
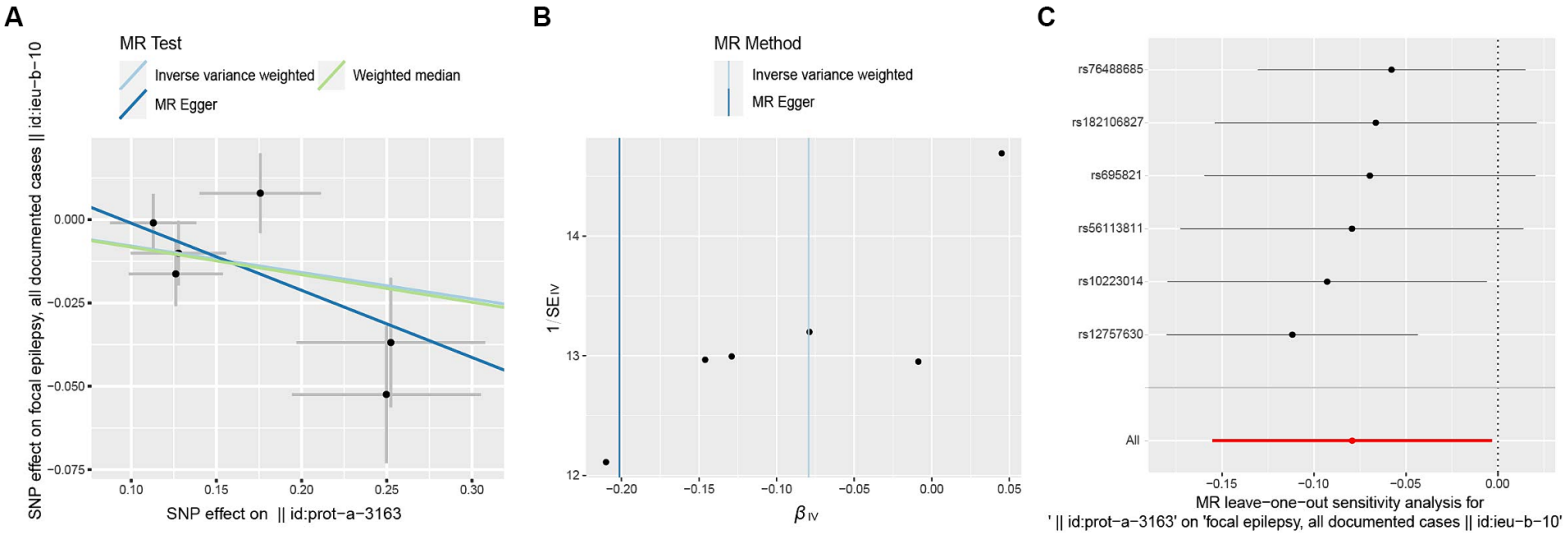
Scatter plot **(A)**, funnel plot **(B)**, leave-one-out analysis **(C)** of the suggestive causal effect of ULK3 on focal epilepsy.

The F-statistic for individual SNPs ranged from 19.98 to 28.98, suggesting that the causal association was unlikely to be affected by weak instrumental variable bias (Supplementary Table 1). Using the IVW method, we found a significant association between ULK3 and an increased risk of focal epilepsy (odds ratio (OR) = 0.924, 95% confidence interval (95% CI): 0.622–0.856, *p* = 0.041). The MR-Egger, weighted median, and weighted model methods yielded more conservative estimates that did not reach statistical significance (Figure 2). No evidence of pleiotropy (intercept = 0.019, *p* = 0.414) or heterogeneity (*p* = 0.165) was observed. Scatterplots and funnel plots displayed a symmetrical distribution of points of causal effects, suggesting minimal susceptibility to potential bias. Additionally, the LOO analysis found no significant disproportionate effect for any SNP on the causal estimates (Figure 3; Table 1).

**Table 1:** Heterogeneity and pleiotropy tests for bidirectional TSMR analyses between ULK3 and epilepsy.

Exposure	Outcome	Q-value (IVW)	P _Q_ (IVW)	Q-value (MR-ER)	P _Q_ (MR-ER)	Intercept	P _Intercept_
ULK3	Focal epilepsy	7.8463	0.1649	6.5014	0.1647	0.0191	0.4145
ULK3	Generalized epilepsy	4.4489	0.4868	4.4101	0.3533	0.0047	0.8604
Focal epilepsy	ULK3	50.1598	0.3878	48.1278	0.4270	0.0205	0.1655
Generalized epilepsy	ULK3	64.4024	0.4273	64.0974	0.4028	0.0108	0.5889

Q-value, the statistics of Cochrane’s Q test; P Q, *p*-value corresponding to Cochrane’s Q test; P intercept, *p*-value corresponding to MR-Egger intercept test; TSMR, Two Sample Mendelian randomization.

### 3.3 ULK3 and generalized epilepsy

**Figure 4:**
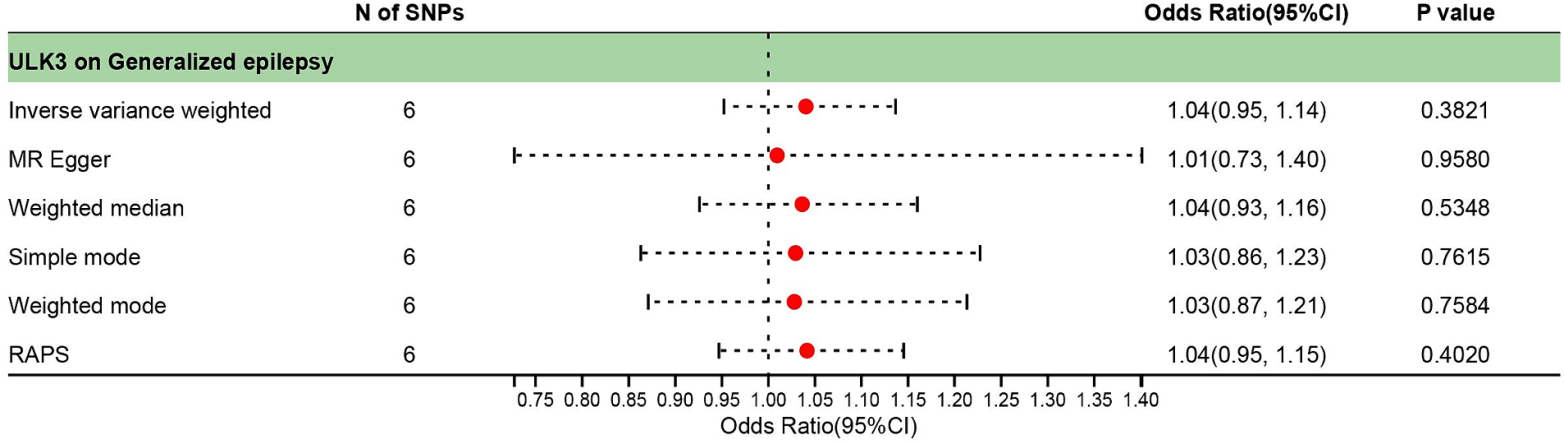
Forest plot of causal effect between ULK3 and generalized epilepsy.

**Figure 5:**
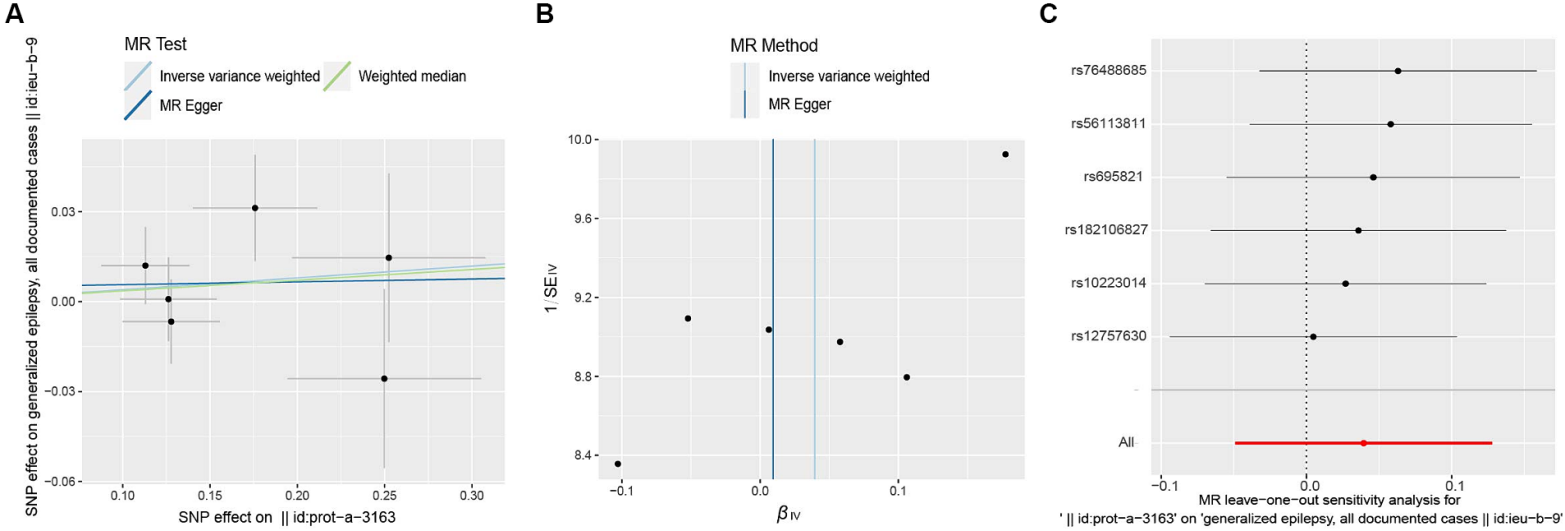
Scatter plot **(A)**, funnel plot **(B)**, leave-one-out analysis **(C)** of the suggestive causal effect of ULK3 on generalized epilepsy.

The F-statistic of individual SNPs ranged from 19.98 to 28.98 (Supplementary Table 2). Using IVW, MR-Egger, weighted median, and weighted model methods, no significant causal relationship was found between ULK3 and the risk of generalized epilepsy (OR = 1.04, 95% CI: 0.727–0.952, *p* = 0.382; Figure 4). No evidence of pleiotropy (intercept = 0.005, *p* = 0.860) or heterogeneity (*p* = 0.487) was detected (Figure 5; Table 1).

### 3.4 Reverse MR analysis

**Figure 6:**
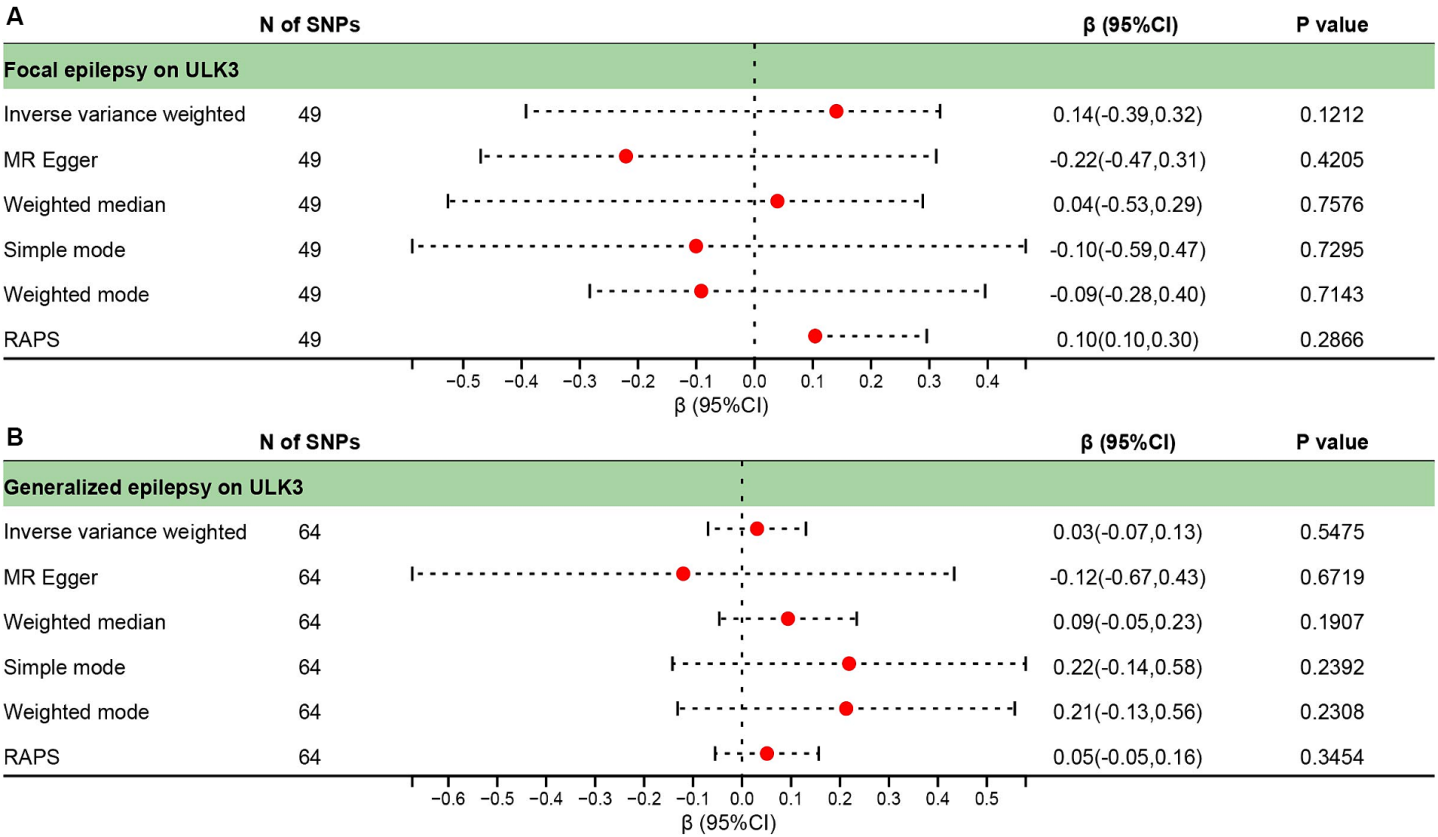
Forest plot of reverse causal effect between ULK3 and focal epilepsy **(A)**, ULK3 and generalized epilepsy **(B)**.

**Figure 7:**
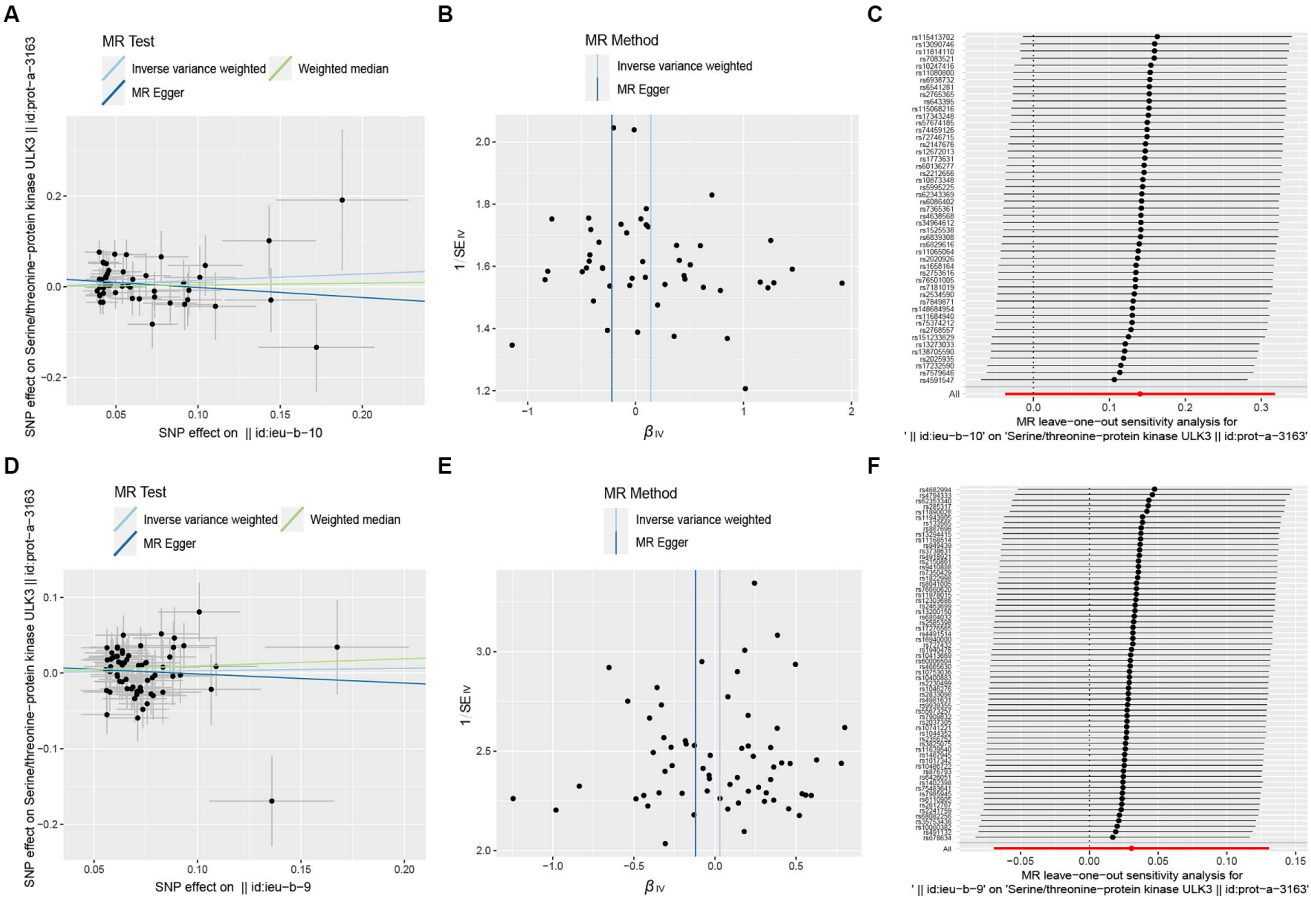
Scatter plot **(A,D)**, funnel plot **(B,E)**, leave-one-out analysis **(C,F)** of the suggestive causal effect of ULK3 on focal and generalized epilepsy, respectively.

To investigate whether focal epilepsy and generalized epilepsy had any causal effect on ULK3, a reverse MR analysis was conducted. By identifying SNPs closely associated with epilepsy as genetic tools for exposure, the F-statistic for epilepsy was well above 10 (focal epilepsy: 33.55, generalized epilepsy: 71.76; Supplementary Tables 3, 4), indicating no evidence of weak instrument bias. Focal epilepsy (OR = 1.15, 95% CI: 0.471–0.963, *p* = 0.121) and generalized epilepsy (OR = 1.03, 95% CI: 0.510–0.933, *p* = 0.548) showed no significant causal effect on ULK3 (Figures 6, 7; Table 1).

## 4 Discussion

In this study, we leveraged subtype classification data from a substantial sample size within the 2018 International Union Against Epilepsy (ILAE) to enhance the reliability of our subtype-specific data. Using bidirectional MR analysis, we explored the association between epilepsy and ULK3. In the forward MR analysis, we observed that a higher serum level of ULK3 was associated with a protective effect against focal epilepsy. However, we found no causal relationship between ULK3 and generalized epilepsy.

Recently, the role of ULK3 in epilepsy development remains unclear. There is no significant relationship has not been reported previously. ULK3 positively regulates SHH signaling as a pathway regulator (32). The SHH signaling pathway is crucial for axon formation, proliferation, survival, and differentiation (33). SHH can inhibit the activity of glutamate transporters in neurons (6), potentially impacting the development of epileptic-like activity. Recent studies (5) have demonstrated that ULK3 is involved in autophagy as a positive regulator of autophagy. The elevation of autophagy-related proteins is associated with neuronal plasticity and epileptic behavior (34).

Epilepsy represents a multifaceted neurological disorder characterized by the involvement of multiple genes, proteins, and signaling pathways in its pathogenesis. Among these contributing factors, ULK3 is identified as potentially influential, particularly within specific types of epilepsy. It is important to recognize that distinct types of epilepsy may engage varying genetic, molecular, and cellular mechanisms. Prior studies have postulated that the differentiation between generalized epilepsy and focal epilepsy manifests as variances in the brain structure of affected individuals. In cases of generalized epilepsy, the epileptogenic network implicates bilateral thalamic cortical structures and exhibits widespread distribution throughout the brain (35), often linked to genetic factors. Conversely, focal epilepsy entails neural circuits within the cerebral hemispheres, typically the neocortex or limbic cortex, which could be incited by local injury, inflammation, tumors, or other brain abnormalities (36). Notably, focal seizures commonly manifest as a primary symptom in patients with glioma (37). Consequently, epilepsy emerges as a diverse condition influenced by a spectrum of genetic, environmental, and physiological factors. While elevated levels of ULK3 may correlate with reduced susceptibility to partial epilepsy in certain individuals, it is essential to acknowledge that other factors, such as additional genetic components and environmental influences, may exert a more substantial influence on the risk of generalized epilepsy, thus resulting the causative association of ULK3 inconclusive.

However, our study has several limitations. The study population consisted mainly of individuals of European ancestry, which may limit the generalizability of our findings to other populations. Additionally, the subtypes we studied, generalized epilepsy and focal epilepsy, had relatively small case numbers, necessitating analysis of larger sample sizes in future research to increase result confidence. In addition, the GWAS data in this study are based on results from a cross-sectional study, and longitudinal analysis of ULK3 and epilepsy is required to confirm our hypothesis. Besides, this study includes circulating levels of ULK3 protein, not protein levels in the brain or cerebrospinal fluid, which may be indirect for the assessment of epilepsy risk. Unmeasured and residual confounding factors that have not been accounted for may introduce bias into the overall outcome estimate.

## 5 Conclusion

This study marks the first exploration of a causal relationship between ULK3 and focal epilepsy. It contributes to our understanding of the pathological basis of epilepsy. Our findings provide valuable insights and impetus for further research.

## Data availability statement

The original contributions presented in the study are included in the article/Supplementary material, further inquiries can be directed to the corresponding author.

## Ethics statement

Ethical review and approval was not required for the study on human participants in accordance with the local legislation and institutional requirements. Written informed consent from the patients/participants or patients/participants' legal guardian/next of kin was not required to participate in this study in accordance with the national legislation and the institutional requirements.

## Author contributions

BL: Writing – review & editing, Supervision, Formal analysis, Conceptualization. KF: Writing – original draft. XZ: Writing – review & editing, Data curation. YZ: Writing – review & editing, Data curation. SB: Writing – review & editing, Data curation. ZL: Writing – review & editing, Data curation. SX: Writing – review & editing, Data curation. ZS: Writing – review & editing, Data curation. HC: Writing – review & editing, Methodology. HZ: Writing – review & editing, Methodology. SZ: Writing – review & editing.

## Footnotes

^1^https://www.phpc.cam.ac.uk/ceu/proteins
